# Comparative Adhesion, Ageing Resistance, and Surface Properties of Wood Plastic Composite Treated with Low Pressure Plasma and Atmospheric Pressure Plasma Jet

**DOI:** 10.3390/polym10060643

**Published:** 2018-06-09

**Authors:** Andrés Jesús Yáñez-Pacios, José Miguel Martín-Martínez

**Affiliations:** Adhesion and Adhesives Laboratory, Department of Inorganic Chemistry, University of Alicante, 03080 Alicante, Spain; andresjesus.yanez@ua.es

**Keywords:** wood plastic composite, surface treatment, low pressure plasma, atmospheric pressure plasma jet, surface energy, adhesion, ageing

## Abstract

Wood plastic composites (WPCs) have poor adhesion properties due to their high surface concentration in non-polar polymers. In this work, two different plasma surface treatments, low pressure plasma (LPP) and atmospheric pressure plasma jet (APPJ), are proposed to increase the surface energy and adhesion property of WPC made with polyethylene (PE-WPC). After optimizing the conditions for each plasma surface treatment, the surface modifications and adhesion of PE-WPC treated with LPP and APPJ were compared. The optimal surface modifications of PE-WPC were obtained by treatment with Argon (Ar): Oxygen (O_2_) LPP for 90 s, and with air APPJ by using a plasma nozzle-WPC surface distance of one centimeter and speed of platform of one meter per minute. Both plasma treatments produced similar chemical modifications and surface energies on the PE-WPC surface. The ablation was more important for Ar:O_2_ LPP treatment, and the air APPJ treatment produced more extensive chemical modifications and more homogeneously removal of the wood component of the surface, rendering the polymer surface smoother. Adhesion of PE-WPC was similarly improved by treatment with both plasmas, from 56 N/m in the as-received to 92–102 N/m in the plasma treated PE-WPC joints. The influence of ageing at 24 °C and 40% relative humidity of the adhesive joints made with PE-WPC surface and treated with Ar:O_2_ LPP and APPJ plasmas was studied. In the joints made with plasma-treated PE-WPC aged under open air for more than one day, the adhesion decreased. An adhesive strength near to that of the joint made with the as-received PE-WPC was obtained after six days. However, if the adhesive joint was created immediately after plasma treatment and peeled at different times, the adhesion was maintained and even increased, and the hydrophobic recovery of the plasma-treated PE-WPC surface was inhibited.

## 1. Introduction

Wood plastic composites (WPCs) are alternative materials to wood, with improved mechanical properties and enhanced outdoor resistance. WPCs are made of wood flour, polymer matrix, and additives, such as lubricants, biocides, and stabilizers. The polymer is added in amounts of 30 to 70 wt %; the most common polymers are polyethylene, polypropylene, or poly (vinyl chloride) [1]. The additive content is usually lower than five wt % and they are intended to increase the compatibility between the polar wood flour and the non-polar polymer components of the WPC.

During processing, the surface of the WPC is enriched with non-polar polymer and, consequently, its surface energy is low and its adhesion is poor. Due to the poor adhesion of WPCs, they cannot be joined with adhesives and they cannot be painted, colored, or decorated with paints or coatings. The actual joining procedures of WPC include mechanical interlocking, anchoring to metallic structural frame, and the use of nails. These procedures are limited in WPCs with complicated geometric shapes, and the use of adhesives would solve this limitation. Furthermore, WPCs are currently colored by adding the dye or the colorant during WPC processing, complicating the creation of solid colors [2]. The application of surface treatments can increase the adhesion of WPC to adhesives and coatings.

Several physical and chemical surface treatments have been proposed in the literature to improve the surface energy and/or the adhesion of different WPCs [3], including immersion in chromic acid, mechanical abrasion, and treatment with flame [4,5,6,7]. The surface treatment of WPCs with chromic acid was the most effective due to the chemical modifications produced on the surface, but this surface treatment is not environmentally friendly so new surface treatments must be developed. Gramlich et al. [1] established that surface treatment with flame, water, or combinations of both improved the shear strength of adhesive joints made with WPC made with polyolefin but not as much as achieved with chromic acid treatment. Yáñez-Pacios and Martín-Martínez [8] proposed surface treatment with ultraviolet (UV) and ozone for improving the surface energy and peel strength of WPC. The increase in adhesion was more noticeable by increasing the UV radiation dose.

Because WPCs are made of wood flour and polyolefin, during processing, their outermost surfaces are enriched in the polyolefin component. Therefore, for improving the adhesion properties of the WPCs, the same surface treatments for improving the adhesion of polyolefin can be applied. The most relevant existing literature has demonstrated that surface treatments with low pressure and atmospheric plasmas were the most effective for improving the adhesion of polyethylene and polypropylene polymers [9,10,11,12,13,14,15,16]. Both nitrogen and oxygen low-pressure plasmas have been successfully used for improving the adhesion of polyethylene polymer [10,11,12], which was mainly ascribed to surface oxidation. Different atmospheric pressure plasmas have been proposed for improving the adhesion of polyethylene and polypropylene polymers [13,14,15,16] and the improved adhesion was ascribed to the creation of a carbon-oxygen polar group on the polyolefin surface. Therefore, surface treatment with low pressure and/or atmospheric pressure plasmas should also increase the adhesion of WPC. In fact, some studies have demonstrated the usefulness of the plasma treatment for improving the surface energy and polarity of different WPCs but most of them did not consider the adhesion property.

Different atmospheric pressure plasma surface treatments for WPCs have been proposed in the literature. Moghadamzadeh et al. [6] used corona discharge surface treatment to effectively improve the surface polarity and shear strength of joints made with high-density polyethylene-based WPC and two-component epoxy adhesive. Along the same lines, Akhtarkhavari et al. [7] proposed the use of corona discharge surface treatment for improving the paintability and the adhesion of WPC made with polyethylene. Several additional studies confirmed that corona discharge surface treatment improved the adhesion of WPCs, mainly due to surface oxidation [17,18,19]. However, this treatment was not effective in avoiding hydrophobic recovery [17], as the surface modifications on the WPC surface were lost over time and the shear bonding strength decreased with ageing. Other kinds of atmospheric pressure plasmas have been proposed for the surface treatment of WPCs. Wolkenhauer et al. [20] treated polyethylene-based WPC with dielectric barrier discharge to produce chemical modifications, increase surface energy, and improve adhesion. Liu et al. [21] and Hünnekens et al. [22] obtained similar results, confirming the effectiveness of atmospheric plasmas on the improvement of the adhesion of WPC. Different air- or oxygen-forced atmospheric plasmas have been proposed for improving the surface properties of polyethylene and polypropylene based WPCs [23,24,25], and enhanced wettability and improved adhesion were obtained, irrespective of the polymer matrix in the WPC.

Low pressure plasma surface treatments for WPCs have scarcely been studied in the period 2008 to 2018. Gupta and Laborie [26] compared the effectiveness of several surface treatments for the surface activation of different WPCs, and they found that the oxygen low pressure plasma surface treatment notably improved the initial adhesion. Furthermore, the treatment with oxygen low-pressure plasma improved the wettability, enhanced the surface roughness, and increased the interfacial adhesion of the WPCs. Air or oxygen low-pressure plasma surface treatments produced crosslinking, chain scission, ablation, oxidation, and even surface roughening of polymers and WPCs, and these surface modifications contributed to the improvement of their adhesion [12,26,27]. In another study, Yáñez-Pacios and Martín-Martínez [28] used Ar:O_2_ low-pressure plasma for improving the surface properties and adhesion of polyethylene-based WPC. 

The surface modifications on polyethylene produced by low pressure plasma have been shown to be lost by increasing the time after treatment due to ageing and/or hydrophobic recovery [29,30,31]. Several publications [32,33,34] studied the stability of surface modifications on different non-polar polymers treated with low-pressure plasma. They concluded that immersion in polar liquids and short-term treatment increased the stability of the surface modifications with time. However, to the best of our knowledge, the stability of the surface modifications of low-pressure plasma-treated WPCs has not yet been studied. On the other hand, only one study [35] addressed the ageing of atmospheric pressure plasma-treated WPCs made with polyethylene and polypropylene. Ageing studies were completed by storing the surface-treated WPC pieces at different temperatures (20 or 60 °C) and relative humidity (0, 65, and 75%). They concluded that ageing time was not the main parameter determining ageing; the relative humidity and the temperature affected more the loss of the surface modifications over time. On the other hand, the increase in the time between the surface treatment and the coating or gluing of the treated WPCs caused a decrease in the wettability [35].

Although some publications are related to the effects produced by the surface treatment of WPCs with different kind of plasmas, neither a comparison between the effectiveness of treatment with low-pressure and atmospheric pressure plasmas of WPCs, nor a comparative study of the influence of ageing with time on their adhesion, have been performed. Therefore, in this work, two different plasma surface treatments (Ar:O_2_ low pressure plasma and air atmospheric pressure plasma jet) of WPC made with polyethylene were optimized and compared, and the changes in the surface properties, topography, adhesion, and ageing resistance over time were studied.

## 2. Materials and Methods

### 2.1. Wood Plastic Composite

Commercial high-density polyethylene-based WPC (PE-WPC) made by extrusion was used. PE-WPC was supplied by Condepols (Jaén, Spain) in the form of alveolar boards that were cut into pieces of 3 × 7 cm^2^ for surface treatment and characterization. PE-WPC has high wood content (43 wt % obtained by thermal gravimetric analysis experiments [25]). 

### 2.2. Plasma Surface Treatments

#### 2.2.1. Ar:O_2_ Low Pressure Plasma (LPP)

**Figure 1:**
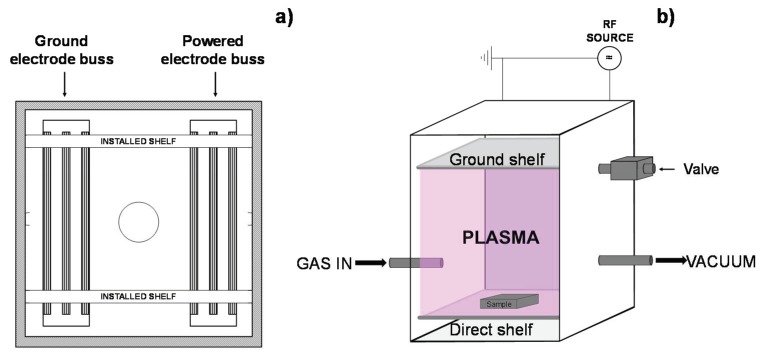
Scheme of low-pressure plasma (LPP) reactor used for surface treatment of polyethylene wood plastic composite (PE-WPC): (**a**) chamber scheme and (**b**) direct configuration scheme.

PE-WPC was surface treated with radiofrequency (13.56 MHz) low-pressure plasma in a Digit Concept NT1 (BSET EQ, Antioch, CA, USA) plasma reactor. The plasma reactor is made of stainless steel chamber with aluminum shelves. The side walls of the plasma reactor have shelf guides to slide the shelves into the back tracks. Brass and aluminum electrode buss bars are placed on the back wall of the plasma reactor, the power shelf is plugged into the brass electrode bar, and the ground shelf is plugged into the aluminum buss electrode (Figure 1). Thus, depending on the connection of the power and ground shelves into the electrode buss bars, different configurations of the plasma reactor can be obtained. The “direct” configuration of the shelves, in which the PE-WPC is placed over the power shelf that is located below the ground shelf, was used in this study. A mixture of argon:oxygen (2:1, vol/vol; oxygen flow: 50 standard cm^3^/min; argon flow: 110 standard cm^3^/min) was used to generate the plasma. This specific gas combination was selected due to its effectiveness in avoiding the surface migration of low molecular weight species as demonstrated in previous studies [28,36]. Ar:O_2_ LPP should produce an adequate balance between the chemical (mainly oxidation) and physical (mainly ablation) modifications produced on the surface of different polymers. The power was set to 200 W, the residual pressure was 800 mbar, and the treatment time ranged between 20 and 90 s. Because PE-WPC contains relatively significant amounts of moisture, to avoid the influence of the desorbing species, including water and additives, from the PE-WPC during LPP treatment, the residual pressure used in this study was higher than usual.

#### 2.2.2. Air Atmospheric Pressure Plasma Jet (APPJ)

**Figure 2:**
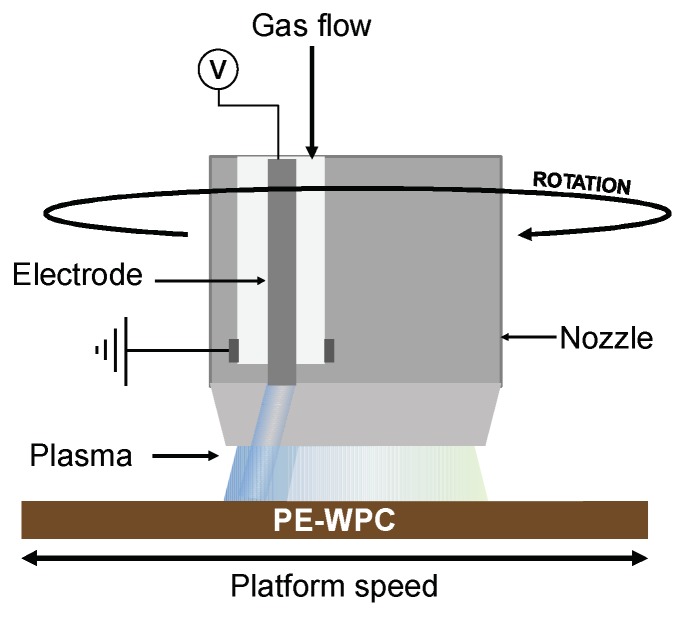
Scheme of atmospheric pressure plasma jet (APPJ) system used for the surface treatment of PE-WPC.

PE-WPC was surface treated in an APPJ Openair FG1001 plasma generator (Plasma Treat GmbH, Steinhagen, Germany) with a RD1004 rotary nozzle with an opening ring of 4 mm diameter. The plasma was generated inside the nozzle by a non-equilibrium discharge using kHz excitation (voltage of 300 V and current of 8.6 A) and expelled through a circular orifice onto the PE-WPC surface. The PE–WPC sample was placed on an electronically controlled speed platform placed below the plasma jet (Figure 2). Compressed synthetic air (Air Liquide, Madrid, Spain) was used for generating the plasma, the pressure of the compressed air was set to 2.5 bars, and the air flow was 114 normal L/min. The plasma was expelled through the rotary nozzle and the speed of the exit of the plasma was controlled by setting the rotational speed to 1900 rpm and the angle shot to 14°. The PE-WPC surface-nozzle distance was set to 1 cm, and the treatment time was varied by changing the speed of the electronically controlled platform on which the PE-WPC sample was placed, between 1 and 8 m/min.

### 2.3. Characterization Techniques

#### 2.3.1. Attenuated Total Reflectance Infrared Spectroscopy (ATR-IR)

The chemical modifications produced in the PE-WPC surface treated with different plasmas were assessed by attenuated total reflectance infrared spectroscopy (ATR-IR) in an Alpha spectrometer (Bruker Optiks, Etlinger, Germany). A germanium prism was used. The incidence angle of the infrared (IR) beam was 45° and 60 scans were obtained and averaged with a resolution of 4 cm^−1^. Under these experimental conditions, a depth of about 1 μm of the PE-WPC surface was analyzed.

#### 2.3.2. Contact Angle Measurements

The wettability of the as-received and plasma-treated PE-WPC surface was estimated by contact angle measurements at 25 °C in a Ramé-Hart 100 goniometer (Netcong, NJ, USA). Two test liquids of different polarity, bidistilled and deionized water (polar liquid) and diiodomethane (non-polar liquid), were used. Drops of 4 µL of test liquid were placed on the as-received and plasma-treated PE-WPC surface and the contact angles were measured immediately after drop deposition. Due to the surface roughness of the PE-WPC, the tilting plate method was used to measure the advancing and receding angles. Because the contact angle values obtained by the sessile drop method agreed well with the advancing contact angle values, the advancing contact angle values were used. At least five drops of each liquid placed in different locations on the PE-WPC surface were measured and the contact angle values were averaged.

From the advancing contact angle values obtained with water and diiodomethane, the surface energy ($\gamma_{S}$) and their dispersive ($\gamma_{S}^{d}$) and polar ($\gamma_{S}^{p}$) components of the as-received and plasma-treated PE-WPC surface were obtained. The surface energies and their components were estimated by means of Owens-Wendt approach, as shown in Equation (1):

(1) $$(1+\cos\theta_{i})\left(\gamma_{i}^{d}+\gamma_{i}^{p}\right)=2\left(\sqrt{\gamma_{i}^{d}\gamma_{S}^{d}}+\sqrt{\gamma_{i}^{p}\gamma_{S}^{p}}\right)$$

where $\theta_{i}$ is the advancing contact angle value, $\gamma_{i}$ is the surface tension of the test liquid, and the superscripts *p* and *d* indicate the polar and dispersive components of the surface tension of the test liquid or the surface energy of PE-WPC, respectively. The components of the surface tensions of the test liquids were $\gamma_{water}^{p}$ = 51 mN/m and $\gamma_{water}^{d}$ = 21.8 mN/m for water, and $\gamma_{CH_{2}I_{2}}^{p}$ = 0 mN/m and $\gamma_{CH_{2}I_{2}}^{d}$ = 50.8 mN/m for diiodomethane. 

#### 2.3.3. Scanning Electron Microscopy (SEM)

The changes produced by plasma treatments on the PE-WPC topography were assessed with a Jeol JSM-840 microscope (Jeol Ltd., Tokyo, Japan) working at 15 kV. To obtain micrographs with good contrast, prior to being introduced into the SEM chamber, the samples were gold coated in Au/Pd Balzers metallizer SCD 004 (Oerlikon Surface Solutions, Balzers, Liechtenstein, Germany).

#### 2.3.4. Thermogravimetric Analysis (TGA)

The extent of ablation produced by plasma treatment of PE-WPC was quantified by the weight loss produced during the treatment. Thermogravimetric analysis (TGA) was used to determine the variation in weight as a function of the temperature in the as-received and plasma-treated PE-WPC. TGA experiments were carried out using TGA TA Q500 equipment (TA Instruments, New Castle, DE, USA). A total of 10 mg of the sample was placed in platinum crucible and heated from room temperature to 800 °C using a heating rate of 10 °C/min under nitrogen atmosphere (gas flow: 60 mL/min).

#### 2.3.5. Adhesion Measurements

**Figure 3:**
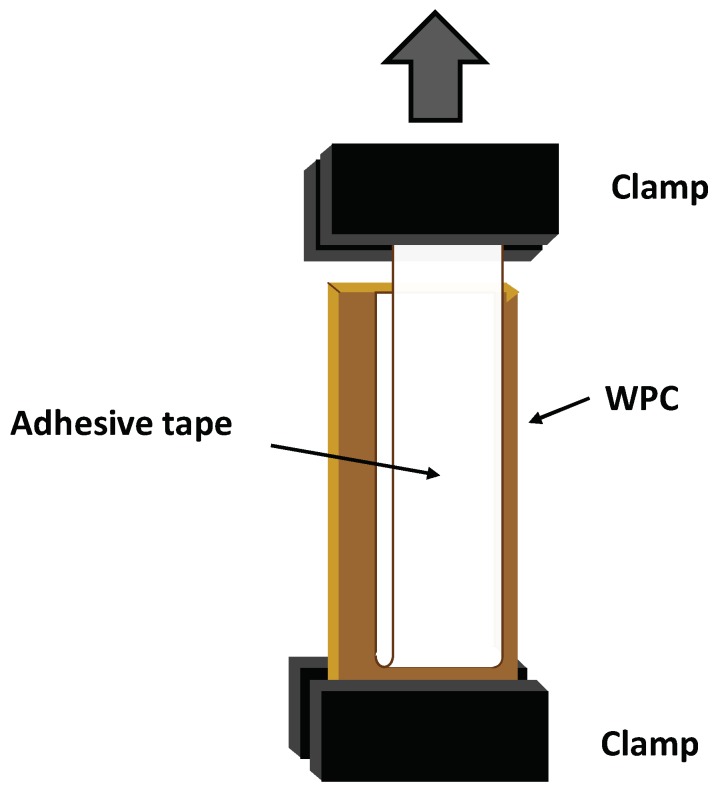
Scheme of test sample for 180° peel test of the PE-WPC/acrylic adhesive tape joint [8].

Adhesive properties were assessed by 180° peel strength of as-received or plasma-treated PE-WPC/Magic Scotch^®^ acrylic adhesive tape joints. Rectangular pieces of PE-WPC, 3 × 7 cm^2^, and rectangular pieces of Magic Scotch^®^ tape (3M, Saint Paul, MN, USA) of 18 × 1.9 cm^2^ were used. The adhesive tape was 11 cm longer than that of PE-WPC to facilitate the attachment to the upper clamp of the test machine for the 180° peel test (Figure 3). Adhesive joints were created by placing the adhesive tape over the PE-WPC, and 30 consecutive passes with 2 kg rubber roller were carried out to create intimate contact between the PE-WPC surface and the acrylic adhesive tape. One hour after joint formation, the 180° peel test was carried out in a TA-XT2i texture analyzer (Stable Micro Systems, Godalming, UK). The peeling rate was 10 mm/s.

**Figure 4:**
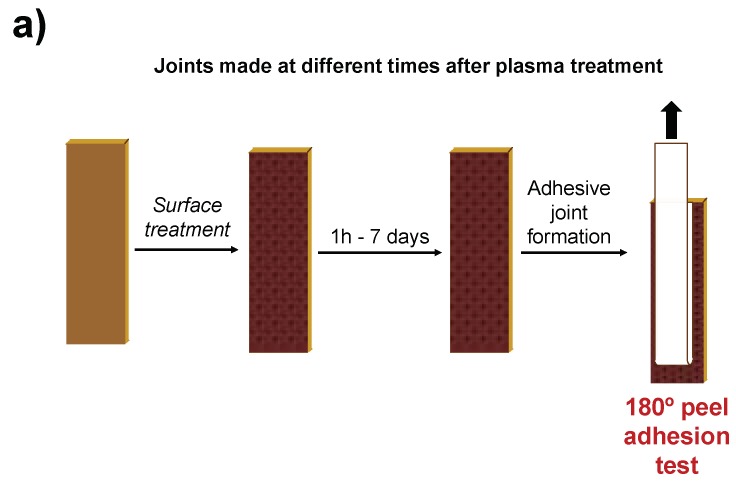

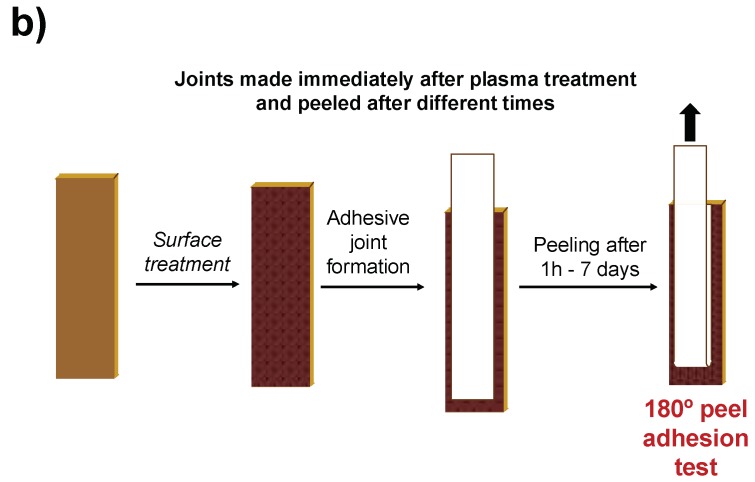
Scheme of the ageing tests of plasma treated PE-WPC/acrylic adhesive tape joints.

The variation in the adhesion of the plasma-treated PE-WPC/acrylic adhesive tape joints was monitored at 24 °C and 40% relative humidity for different times (ageing test). Two different ageing tests were carried out: (1) The plasma-treated PE-WPC was exposed at 24 °C and 40% relative humidity and the joints were created at different times after plasma treatment (Figure 4a). (2) Immediately after treatment, the plasma-treated PE-WPC was joined to the adhesive acrylic tape and left at 24 °C and 40% relative humidity. The 180° peel tests were carried out at different times after joint formation (Figure 4b).

## 3. Results and Discussion

### 3.1. Ar:O_2_ Low Preure Plasma (LPP) Treatment of PE-WPC

**Figure 5:**
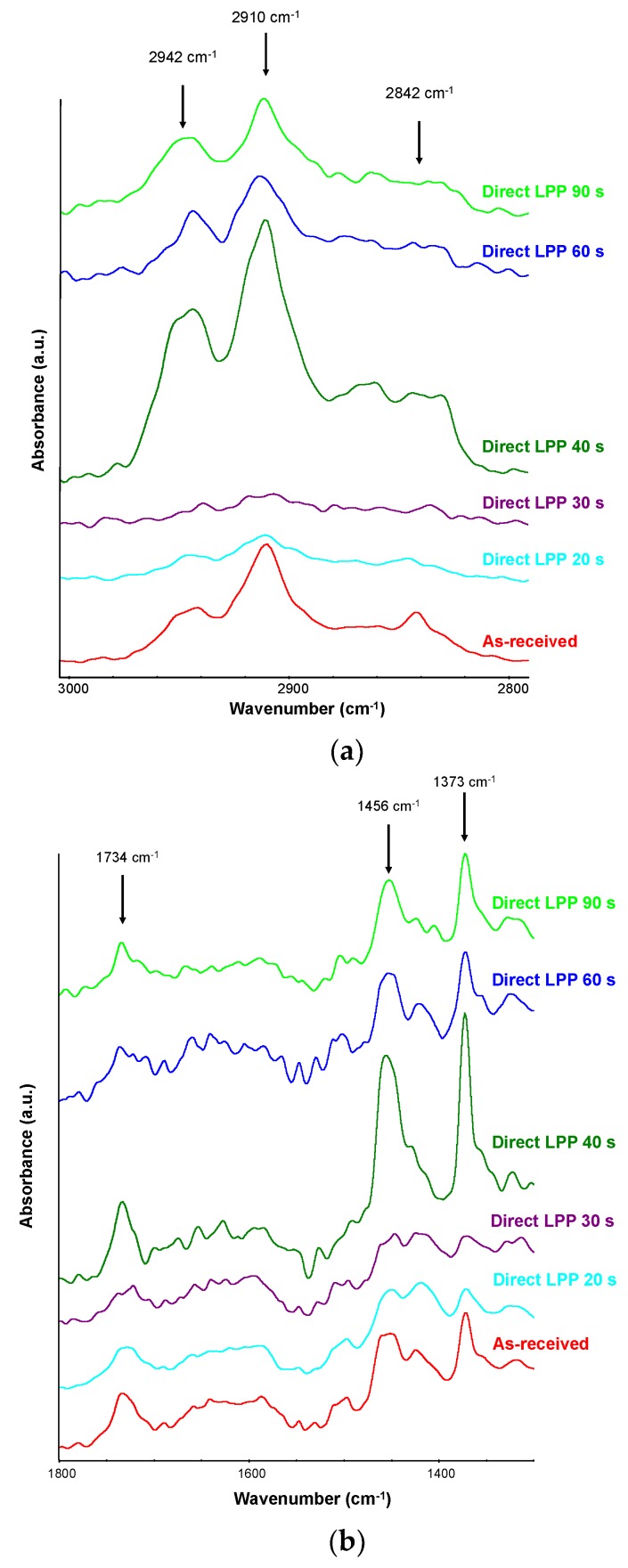
(**a**) Region of 2800–3000 cm^−1^ of the Attenuated Total Reflectance Infrared Spectroscopy (ATR-IR) spectra of the as-received and Ar:O_2_ LPP treated PE-WPC for different times. A germanium prism was used; (**b**) Region of 1300–1800 cm^−1^ of the ATR-IR spectra of the as-received and Ar:O_2_ LPP-treated PE-WPC for different times.

The optimization of the low-pressure plasma surface treatment of PE-WPC was previously studied by Yáñez-Pacios and Martín-Martínez [28], employing direct and secondary downstream shelves configurations. In this study, the direct shelves configuration was used for surface treatment of PE-WPC with Ar:O_2_ LPP and the treatment time varied between 20 and 90 s. The chemical modifications produced by Ar:O_2_ LPP treatment of PE-WPC were assessed by ATR-IR spectroscopy. Figure 5a shows the 2800–3000 cm^−1^ region of the ATR-IR spectra of Ar:O_2_ LPP-treated PE-WPC. The short-term treatment decreases the intensities of the C-H bands at 2842, 2910, and 2942 cm^−1^ due to polyethylene, indicating that the polyethylene was removed from the surface due to the dominance of ablation. However, the increase in the treatment time to 40 s enriched the polyethylene of the treated PE-WPC surface, and for a longer time, similar ATR-IR spectra were obtained. Figure 5b shows the 1300–1800 cm^−1^ region of the ATR-IR spectra of Ar:O_2_ LPP-treated PE-WPC. The short-time treatment decreased the intensity of the polyethylene bands at 1373–1456 cm^−1^ and decreased the intensity of the C=O group at 1734 cm^−1^ due to the wood, indicating the removal of polyethylene and wood material from the surface. The treatment of PE-WPC with Ar:O_2_ LPP for 40 s increased the intensities of both wood and polyethylene bands, and several additional low intensity C=O bands at 1670–1720 cm^−1^ were observed due to polymer oxidation. When the surface treatment was carried out for more than 40 s, all ATR-IR spectra were nearly the same and they showed a lower degree of oxidation because ablation prevailed over the surface oxidation. 

**Figure 6:**
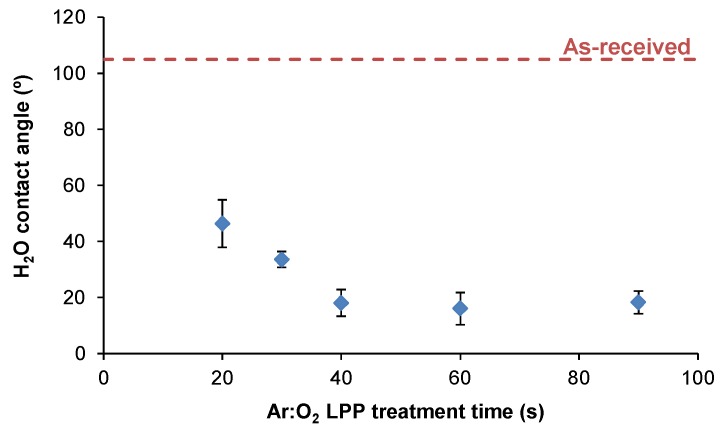
Variation in the water contact angle at 25 °C of the Ar:O_2_ LPP-treated PE-WPC as a function of treatment time. The dashed line corresponds to the water contact angle of the as-received PE-WPC.

The chemical modifications on the Ar:O_2_ LPP-treated PE-WPC surface should modify its wettability. Figure 6 shows the variation in the water contact angle values of the Ar:O_2_ LPP-treated PE-WPC surface as a function of the treatment time. The Ar:O_2_ LPP surface treatment for 20 s noticeably decreased the water contact angle value (from 105° in the as-received PE-WPC to 47°), and the wettability increased more when the treatment was carried out for 40 s. For longer treatment times, similar wettability values (water contact angle of 16–18°) were obtained. Therefore, the Ar:O_2_ LPP treatment for 20 s increased the wettability of the PE-WPC surface due to the removal of polyethylene and the oxidation produced for short times of treatment. For Ar:O_2_ LPP treatment longer than 40 s, ablation was mainly produced. 

**Figure 7:**
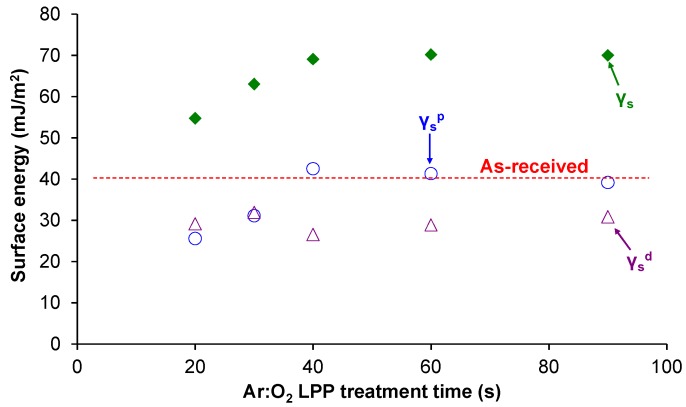
Variation in the surface energy ($\gamma_{s}$) and their polar ($\gamma_{s}^{p}$) and dispersive ($\gamma_{s}^{d}$) components of the Ar:O_2_ LPP-treated PE-WPC as a function of treatment time. The dashed line corresponds to the surface energy of the as-received PE-WPC that only corresponds to the dispersive component.

Figure 7 shows the evolution in the surface energy and its polar and dispersive components of the Ar:O_2_ LPP surface treated PE-WPC as a function of treatment time. The surface energy of the as-received PE-WPC was 39.5 mJ/m^2^ and corresponded to the dispersive component only. The treatment of PE-WPC with Ar:O_2_ LPP increased the surface energy, the increase was mainly due to the polar component. The increase in the surface energy and its polar component were produced up to a treatment time of 40 s; the surface energy was similar for longer times. All Ar:O_2_ LPP surface-treated PE-WPC for different times showed a similar dispersive component of the surface energy. These findings agree well with the results of the ATR-IR spectroscopy.

**Figure 8:**
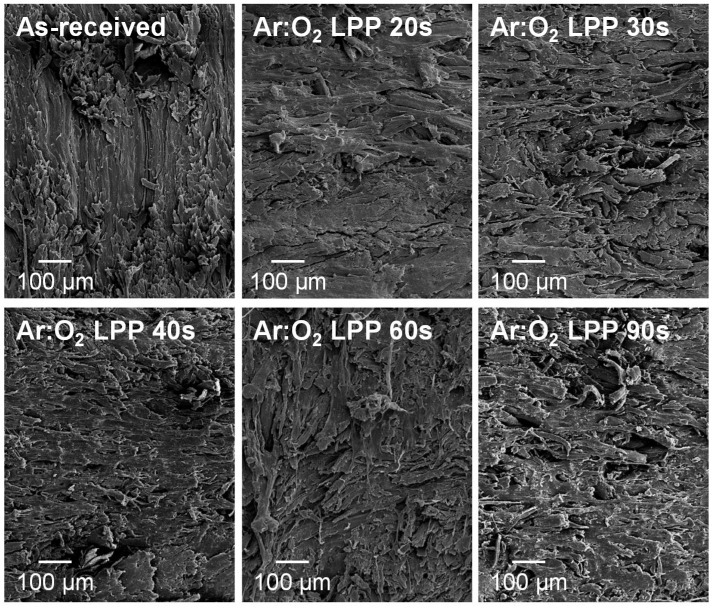
Scanning electron microscopy (SEM) micrographs of the as-received and Ar:O_2_ LPP-treated PE-WPC for different times.

The changes in the topography of the PE-WPC treated with Ar:O_2_ LPP were assessed via SEM microscopy. The SEM micrographs of the as-received and Ar:O_2_ LPP treated PE-WPC are shown in Figure 8. The as-received PE-WPC had a rough surface and the polyethylene component was dominant in the surface. When PE-WPC was treated with Ar:O_2_ LPP for 20 s, ablation was produced and the surface became smoother. The increase in treatment time favored the ablation and the creation of additional new surface roughness and porosity; both were more marked for a treatment time of 90 s. On the other hand, the morphological changes in the Ar:O_2_ LPP-treated PE-WPC cannot be ascribed to thermal damage because the temperature of the just-treated composite was 35–40 °C, which was measured with a non-contact electronic thermometer. 

**Figure 9:**
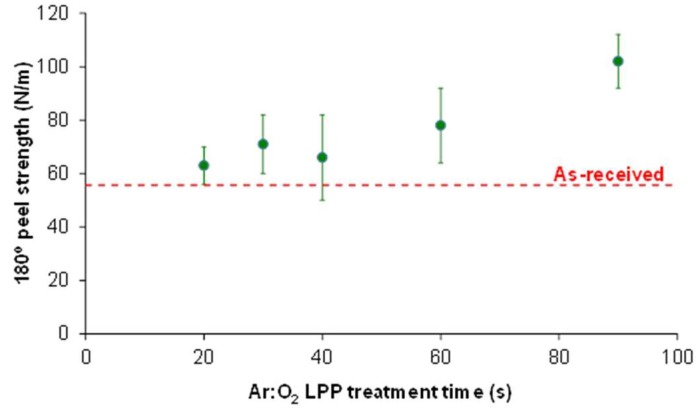
Variation in 180° peel strength of as-received or Ar:O_2_ LPP-treated PE-WPC/acrylic adhesive tape joints as a function of the treatment time. All joints showed adhesion failure.

The 180° peel adhesion tests of the as-received and Ar:O_2_ LPP-treated PE-WPC/acrylic adhesive tape joints were carried out to verify if the surface modifications caused by Ar:O_2_ LPP surface treatment improved the adhesion of PE-WPC. For short treatment times, the adhesion values were close to that of the as-received PE-WPC (56 N/m) (Figure 9), and the increase in the Ar:O_2_ LPP treatment time to 60–90 s produced an increase in adhesion (102 N/m). Therefore, the optimal conditions of the Ar:O_2_ LPP surface treatment of PE-WPC is a treatment time of 90 s.

### 3.2. Air Atmospheric Pressure Plasma Jet (APPJ) Treatment of PE-WPC

A previous study by Yáñez-Pacios and Martín-Martínez [25] showed that a greater distance between the nozzle and the PE-WPC surface was not effective at improving its surface properties. Therefore, in this study, the APPJ treatment of PE-WPC was carried out using a distance of one cm between the nozzle and the PE-WPC surface, varying the platform speed between one and eight m/min. 

**Figure 10:**
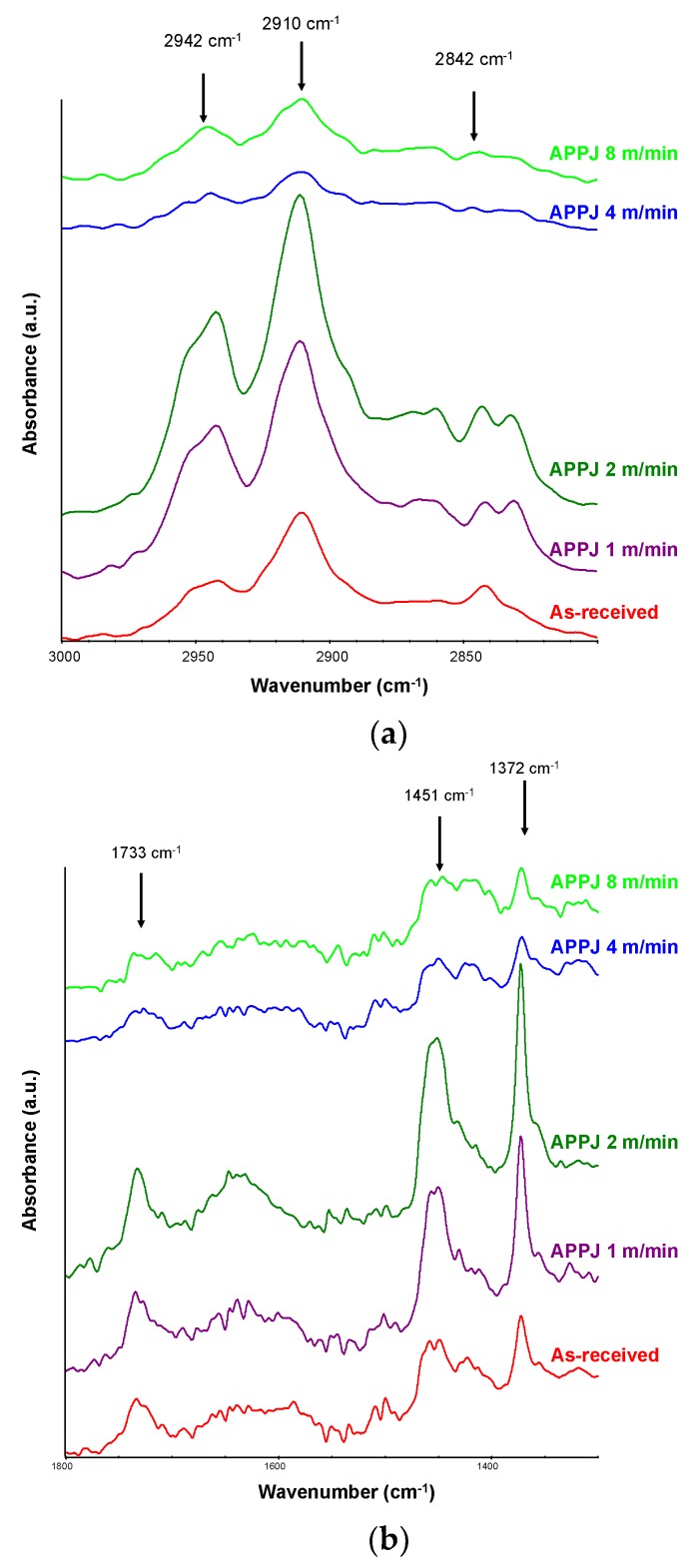
(**a**) Region of 2800–3000 cm^−1^ of the ATR-IR spectra of the as-received and APPJ-treated PE-WPC using different platform speeds. Germanium prism; (**b**) Region of 1300–1800 cm^−1^ of the ATR-IR spectra of the as-received and APPJ treated PE-WPC by using different platform speeds. Germanium prism.

Figure 10a shows the region of 2800–3000 cm^−1^ of the ATR-IR spectra of the as-received and APPJ-treated PE-WPC. More marked chemical modifications were produced for low platform speeds (one to two m/min). The use of platform speeds of four to eight m/min produced an important reduction in polyethylene in the PE-WPC surface, with a noticeable decrease in the intensity of the C-H bands at 2842, 2910, and 2942 cm^−1^, due to slight ablation. An increase in the surface treatment aggressiveness was observed by reducing the platform speed (one to two m/min) producing an increase in the intensity of the polyethylene bands, indicating the removal of the wood component from the PE-WPC surface and the exposure of polymer to the surface. The region of 1300–1800 cm^−1^ in the ATR-IR spectra (Figure 10b) more clearly demonstrates the decrease in the intensities of the polyethylene and wood bands for high-APPJ platform speeds (four to eight m/min), and the increase in the bands of the polyethylene at 1372 and 1451 cm^−1^ and the more intense band of the C=O group at 1733 cm^−1^ due partly to surface oxidation for low-APPJ platform speeds (one to two m/min).

**Figure 11:**
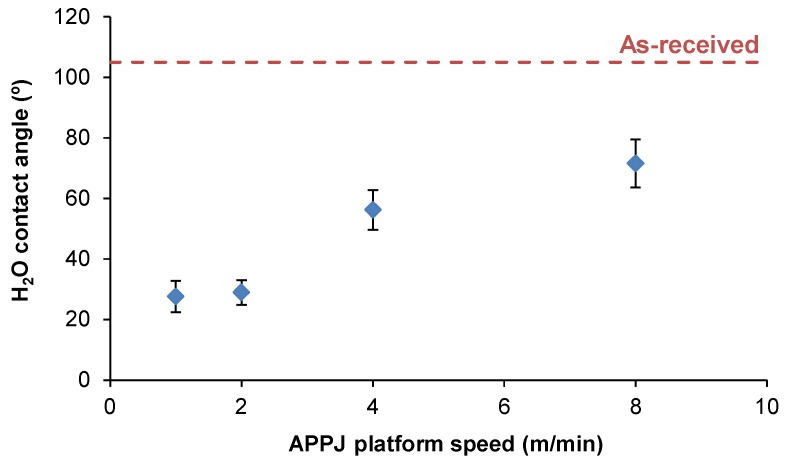
Variation in the water contact angle of the APPJ-treated PE-WPC as a function of the platform speed. The dashed line corresponds to the water contact angle of the as-received PE-WPC.

**Figure 12:**
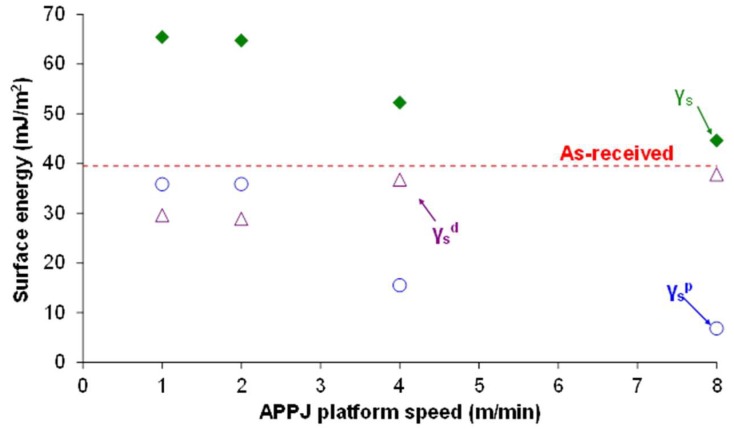
Variation in the surface energy ($\gamma_{s}$) and their polar ($\gamma_{s}^{p}$) and dispersive ($\gamma_{s}^{d}$) components of the APPJ-treated PE-WPC as a function of the platform speed. The dashed line corresponds to the surface energy of the as-received PE-WPC that only corresponds to dispersive component.

Figure 11 shows the evolution of the water contact angle values as a function of the APPJ platform speed. APPJ surface treatment increased the wettability and decreased the water contact angle value of PE-WPC more noticeably by decreasing the platform speed. For platform speeds of one and two m/min, the water contact angle values were low and nearly the same (28–29°), confirming the chemical modifications evidenced by ATR-IR spectroscopy. Similarly, the surface energy of PE-WPC increased with APPJ treatment. The increase was due to the increase of the polar component, which was more marked by decreasing the platform speed or by increasing the treatment time (Figure 12). 

**Figure 13:**
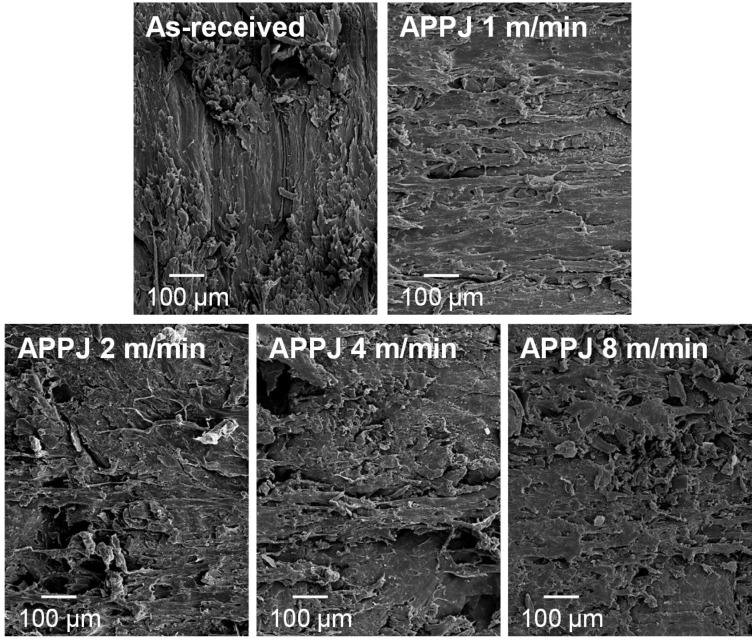
SEM micrographs of the as-received and APPJ-treated PE-WPC with different platform speeds.

The surface roughness of PE-WPC changed with APPJ treatment (Figure 13). The rough surface of the as-received PE-WPC was noticeably reduced by APPJ treatment, and to a greater extent by decreasing the platform speed. Some wood component removal on the PE-WPC surface was produced by treatment with the high speed of the platform, exposing some polymer to the outermost surface. The decrease in the speed of platform enhanced the exposure of polyethylene to the surface and the extent of ablation. On the other hand, the morphological changes on the APPJ-treated PE-WPC cannot be ascribed to thermal damage because the temperature during APPJ treatment was 45–60 °C, which was measured with a non-contact electronic thermometer. Furthermore, the temperature of the just-treated composite was 35–40 °C.

**Figure 14:**
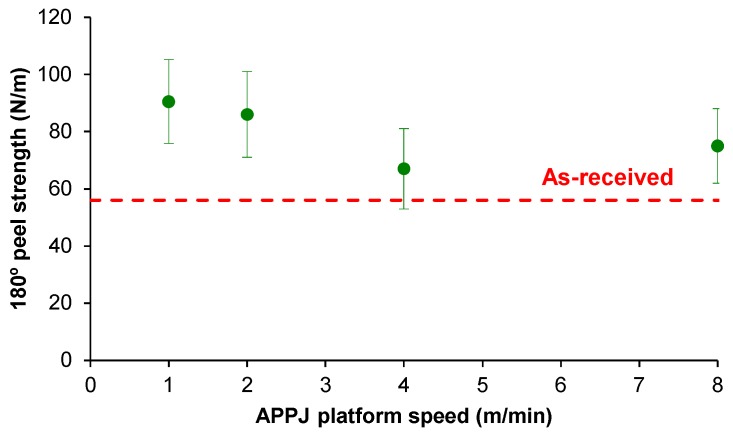
Variation in 180° peel strength of as-received and APPJ-treated PE-WPC/acrylic adhesive tape joints as a function of the platform speed. All joints showed an adhesion failure.

Figure 14 shows the variation in the 180° peel adhesion values for the as-received and APPJ-treated PE-WPC/acrylic adhesive tape joints as a function of the platform speed. For platform speeds of four and eight m/min, the adhesion properties were very similar to that of the as-received PE-WPC, but they improved when the length of the surface treatment increased. For platform speeds of one and two m/min, the highest 180° peel strength was obtained at one m/min. Therefore, the optimal conditions for APPJ treatment of PE-WPC included a composite surface-nozzle distance of one cm and platform speed of one m/min.

### 3.3. Comparison of the Ar:O_2_ LPP and APPJ Treatments of PE-WPC

In this section, the surface modifications and adhesion of PE-WPC treated with plasmas under the optimal conditions are compared. The optimal conditions were Ar:O_2_ LPP treatment for 90 s and air APPJ treatment using PE-WPC surface-nozzle distance of one cm and platform speed of one m/min.

**Figure 15:**
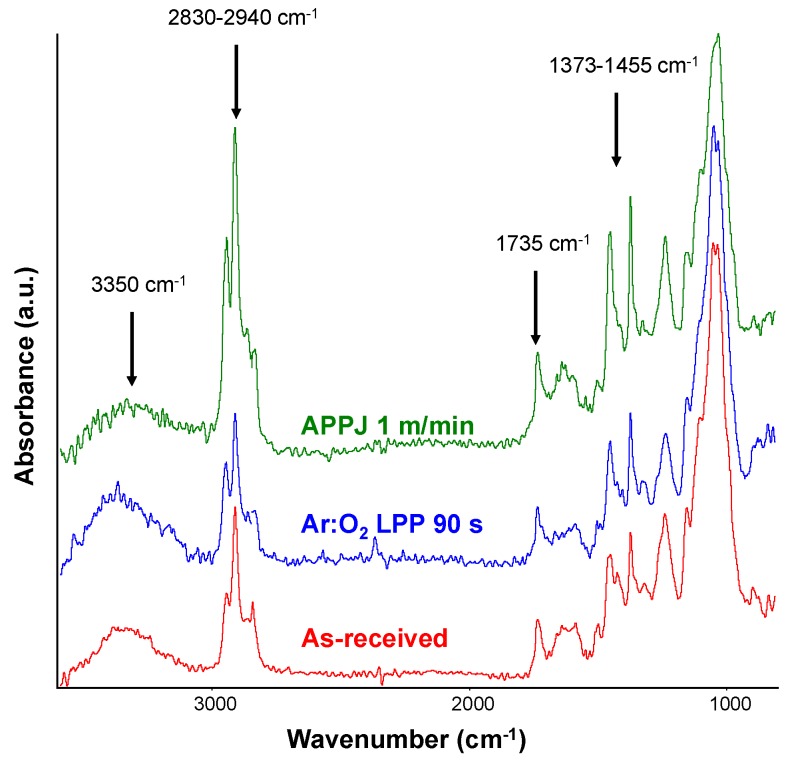
ATR-IR spectra of the as-received and plasma treated PE-WPC. Germanium prism.

Figure 15 shows the ATR-IR spectra of the Ar:O_2_ LPP- and APPJ-treated PE-WPC. The ATR-IR spectra showed that the APPJ treatment produced more marked chemical changes in the PE-WPC surface than Ar:O_2_ LPP treatment, because of the more intense C-H stretching bands at 2830–2940 cm^−1^ and the more intense bands of methylene groups at 1373–1455 cm^−1^, both due to polyethylene. Furthermore, the ATR-IR spectrum of the APPJ-treated PE-WPC showed a lower intensity of the –OH band at 3350 cm^−1^ due to the wood component, and a greater surface oxidation extent, more intense C=O stretching band at 1735 cm^−1^. Therefore, the extent of the chemical modification of PE-WPC is more important when treated with APPJ than with Ar:O_2_ LPP.

**Figure 16:**
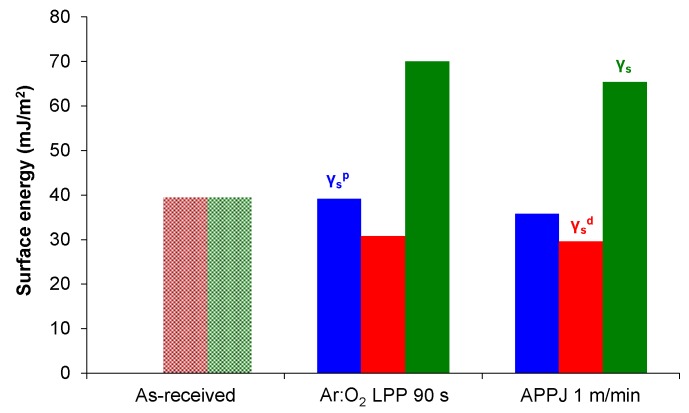
Surface energy and their polar and dispersive components of the as-received and plasma-treated PE-WPC.

The plasma treatment noticeably increased the surface energy of PE-WPC (Figure 16). This increase was due to the polar component. Although the extent of the chemical modifications in Ar:O_2_ LPP-treated PE-WPC were less marked than in APPJ-treated composite, both plasma treatments produced somewhat similar surface energy and similar polar and dispersive components. The Ar:O_2_ LPP treatment exposed less polyethylene polymer to the PE-WPC surface and, therefore, less oxidation was produced. However, the surface energy can also be affected by the changes in the PE-WPC surface topography and roughness produced by both plasmas.

**Figure 17:**
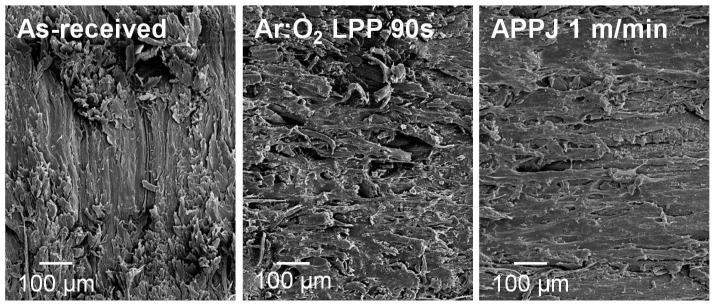
SEM micrographs of the as-received and plasma-treated PE-WPC.

Figure 17 shows the lesser extent of ablation in APPJ-treated PE-WPC than in Ar:O_2_ LPP-treated composite. The roughness of the Ar:O_2_ LPP-treated PE-WPC was more similar to the as-received PE-WPC; different surface topography was obtained in APPJ-treated composite. Therefore, the APPJ treatment removed less wood component from the PE-WPC surface than Ar:O_2_ LPP treatment, which must affect the surface energy.

According to the previous results, the extent of surface oxidation produced on the plasma-treated PE-WPC was less important than the extent of physical ablation. The extent of physical ablation in PE-WPC produced by the plasma treatments was assessed by determining the weight losses produced during the treatment by using a precise analytical balance Cobos AX-200 (Cobos Precisión S.L., Barcelona, Spain) and by thermogravimetric analysis (TGA). The assessment of the extent of ablation of different surface treated polymers from weight loss has been established [37,38]. The PE-WPC pieces were weighted before and after plasma treatment and the weight losses were obtained by averaging the results obtained with five measurements per sample. The weight loss produced in the Ar:O_2_ LPP-treated PE-WPC was 0.59 ± 0.15%, whereas the weight loss of the air APPJ-treated PE-WPC was 0.04 ± 0.01%, confirming the most pronounced ablation of PE-WPC was produced by the Ar:O_2_ LPP treatment. 

**Figure 18:**
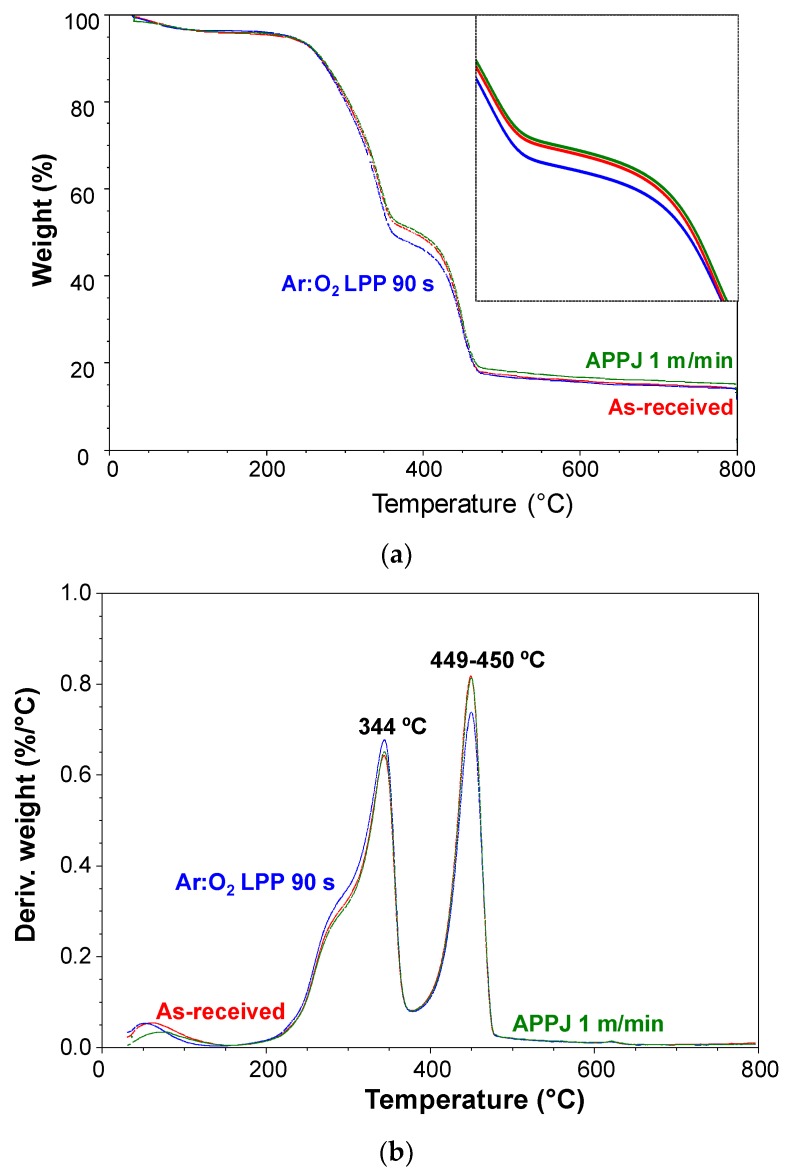
(**a**) Variation in weight as function of the temperature of the as-received and plasma-treated PE-WPC through thermogravimetric analysis (TGA) experiments. (**b**) Variation in derivative of the weight as function of the temperature of the as-received and plasma-treated PE-WPC through TGA experiments.

The extent of ablation of the plasma-treated PE-WPC was also assessed by TGA. Figure 18a shows the variation in weight as a function of temperature for the as-received and plasma-treated PE-WPC. Whereas the TGA thermograms of the as-received and APPJ treated PE-WPC were quite similar, the Ar:O_2_ LPP-treated PE-WPC showed higher weight loss, mainly in the region between 350 and 400 °C. The curves of the derivative of the weight as a function of temperature (Figure 18b) show the existence of three main thermal degradations in the as-received and plasma-treated PE-WPC corresponding to water/moisture loss (maximum temperature of decomposition at 52–78 °C), decomposition of the wood component (maximum temperature of decomposition at 344 °C), and decomposition of the polyethylene component (maximum temperature of decomposition at 449–450 °C). The thermal decompositions of the wood and polyethylene component of the as-received and plasma-treated PE-WPC were produced at the same temperature but the water removal was produced at higher temperatures in APPJ-treated PE-WPC, likely due to the removal of weak hydroxyl groups produced by the treatment. The weight losses corresponding to the three thermal decompositions are provided in Table 1, in which the weight loss due to the wood component is higher and that of the polyethylene component is lower in Ar:O_2_ LPP-treated PE-WPC than in APPJ-treated PE-WPC. Therefore, the more pronounced ablation of Ar:O_2_ LPP-treated PE-WPC was confirmed.

**Figure 19:**
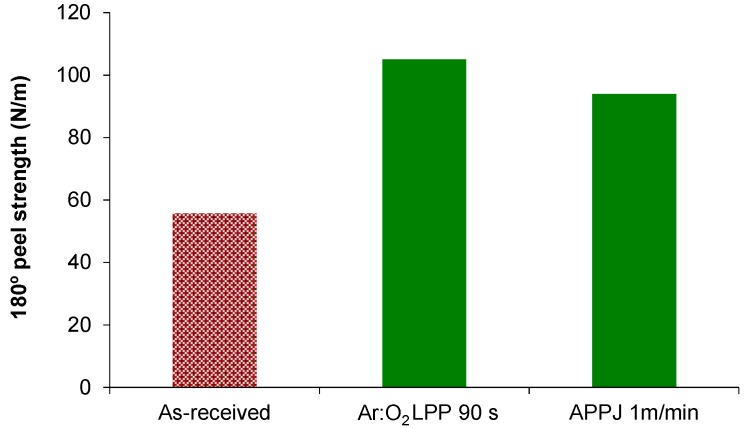
The 180° peel strength values of as-received and plasma-treated PE-WPC/acrylic adhesive joints. An adhesion failure always occurred.

The 180° peel strength values of the as-received and plasma-treated PE-WPC/acrylic adhesive tape joints are shown in Figure 19. The plasma treatment increased the adhesion of PE-WPC; the improvement was somewhat higher when the Ar:O_2_ LPP treatment was used. This slightly higher 180° peel strength can be ascribed to the existence of porosity and the higher roughness of the PE-WPC surface that should increase the mechanical interlocking with the acrylic adhesive tape. In general, similar adhesion was obtained in the joints made with PE-WPC treated with Ar:O_2_ LPP and air APPJ under optimal experimental conditions.

### 3.4. Ageing of Plasma Treated PE-WPC

The surface modifications of the plasma-treated PE-WPC may last over time because of the presence of polyethylene on the outermost surface. The influence of ageing on the adhesion of plasma-treated PE-WPC was studied and two different ageing experiments were carried out. 

**Figure 20:**
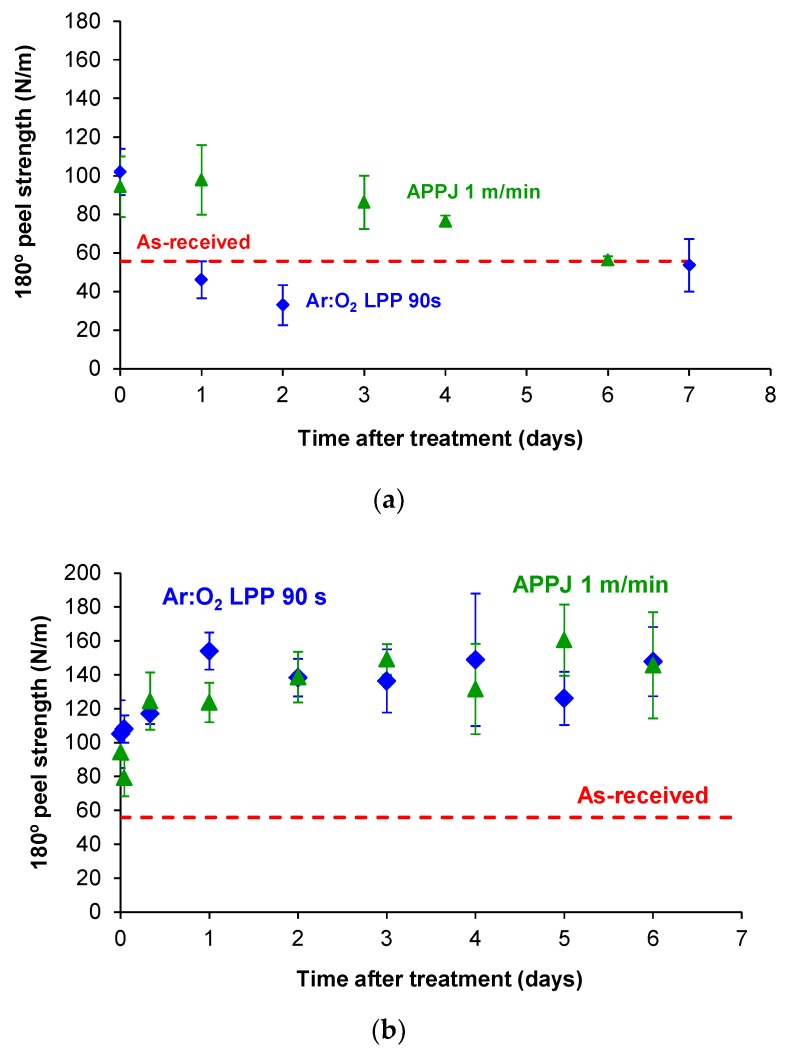
(**a**) Variation in the 180° peel strength of plasma treated PE-WPC/acrylic adhesive tape joints as a function of the time after treatment. Joints were created at different times after plasma treatment and they were tested one hour after joint formation. All joints showed an adhesion failure. The dashed line corresponds to the 180° peel strength of the as-received PE-WPC/acrylic adhesive tape joint. (**b**) Variation in the 180° peel strength of plasma treated PE-WPC/acrylic adhesive tape joints as a function of the time after treatment. Joints were created immediately after plasma treatment and they were tested at different times after joint formation. All joints showed an adhesion failure. The dashed line corresponds to the 180° peel strength of as-received PE-WPC/acrylic adhesive tape joint.

After plasma treatment of PE-WPC, it was left at 24 °C and 40% relative humidity and the adhesive joints were created between one hour and seven days after treatment (Figure 4a). Figure 20a shows the variation in the 180° peel strength as a function of ageing time. During the first day after APPJ treatment, the adhesion of the APPJ-treated PE-WPC was maintained but a gradual decrease in the 180° peel strength was produced by increasing the ageing time. After six days, the 180° peel strength was similar to that of the joint made with the as-received PE-WPC. On the other hand, for the joints created with the Ar:O_2_ LPP-treated PE-WPC, an abrupt decrease in adhesion value was observed one day after treatment, and the adhesion did not vary for longer ageing times, being the same as that of the joint made with the as-received PE-WPC. Therefore, faster hydrophobic recovery was produced in the joints made with Ar:O_2_ LPP-treated PE-WPC, likely due to the lower extent of chemical modifications than in the APPJ-treated composite. After six days, the adhesion of the joints created with the plasma treated PE-WPC was the same and corresponds to the adhesion of the joint made with the as-received PE-WPC, indicating complete hydrophobic recovery, irrespective of the type of plasma used.

Ageing of the plasma-treated PE-WPC was also studied by treating the composite and creating the adhesive joint immediately after plasma treatment; 180° peel tests were carried out at different times (between one hour and six days) after joint formation (Figure 4b). Figure 20b shows that both plasma treatments showed similar variation in the adhesion as a function of ageing time. The adhesion increased up to one day after joint formation and was constant with increasing ageing time thereafter. In fact, an adhesion value of 140 N/m was obtained, irrespective of the kind of plasma treatment. Therefore, hydrophobic recovery was inhibited and even an additional interaction between the polar acrylic tape and the polar surface of the plasma-treated PE-WPC was produced 24 h after joint formation. The nature of these interactions will be investigated in the future.

## 4. Conclusions

The optimal conditions for improving the surface properties and adhesion of PE-WPC were Ar:O_2_ LPP-treatment for 90 s, and air APPJ surface treatment at a distance between the nozzle and the PE-WPC surface of one centimeter, and speed of platform of one meter per minute. 

Both plasma treatments produced similar chemical modifications and caused ablation of the PE-WPC surface. The extent of the chemical modifications was more pronounced by treatment with APPJ and the extent of ablation was more important for treatment with Ar:O_2_ LPP. Furthermore, both plasma treatments modified the surface roughness of the PE-WPC; a smoother surface was produced by treating with APPJ and greater roughness and porosity were created by treatment with Ar:O_2_ LPP. Because of the combination of the chemical modifications and roughness, the surface energies of the plasma-treated PE-WPC increased noticeably and they were similar when they were treated with APPJ and Ar:O_2_ LPP under optimal conditions. The increase was mainly due to the increase in the polar component. Consequently, the 180° peel strength of the joints made with plasma-treated PE-WPCs notably increased from 56 N/m for the joint made with the as-received PE-WPC to 92–102 N/m for the joints made with plasma-treated PE-WPCs. Similar improvements in adhesion were obtained by treatment with APPJ and Ar:O_2_ LPP.

Ageing of the adhesive joints over time was studied by surface treatment of PE-WPC with plasmas, allowing the treated surface to be exposed at 24 °C and 40% relative humidity for different times. Although adhesion was maintained in the joints made with APPJ-treated PE-WPC for one to three days, an important decline was observed after one day ageing in the joints made with Ar:O_2_ LPP-treated PE-WPC. After six days, similar adhesion for the joint made with the as-received PE-WPC was obtained, indicating that full hydrophobic recovery was produced, irrespective of the kind of plasma used. If the adhesive joints were made with the just plasma-treated PE-WPC, one day after joint formation, the adhesion increased to 140 N/m, similarly for the joints made with Ar:O_2_ LPP and APPJ, and the 180° peel strength was maintained up to six days. Therefore, hydrophobic recovery was completely avoided.

## Figures and Tables

**Table 1:** Weight losses of the thermal decompositions of the as-received and plasma treated polyethylene wood plastic composite (PE-WPC) obtained with thermogravimetric analysis (TGA) experiments.

Surface Treatment	Weight Loss (%) at 52–78 °C	Weight Loss (%) at 344 °C	Weight Loss (%) at 449–450 °C
As-received	4	45	35
Ar:O_2_ LPP 90 s	3	49	32
APPJ 1 m/min	3	45	35
